# Neural Correlates of Stepping in Healthy Elderly: Parietal and Prefrontal Cortex Activation Reflects Cognitive-Motor Interference Effects

**DOI:** 10.3389/fnhum.2020.566735

**Published:** 2020-09-29

**Authors:** Julia Reinhardt, Oana G. Rus-Oswald, Céline N. Bürki, Stephanie A. Bridenbaugh, Sabine Krumm, Lars Michels, Christoph Stippich, Reto W. Kressig, Maria Blatow

**Affiliations:** ^1^Department of Neuroradiology, Clinical Neuroscience Center, University Hospital Zurich, University of Zurich, Zurich, Switzerland; ^2^Department of Radiology, Division of Diagnostic and Interventional Neuroradiology, University Hospital of Basel, University of Basel, Basel, Switzerland; ^3^University Department of Geriatric Medicine Felix Platter, Basel, Switzerland

**Keywords:** gait, elderly, cognitive-motor, dual task, interference

## Abstract

Gait analysis involving cognitive-motor dual task (DT) is a diagnostic tool in geriatrics. Cognitive-motor interference effects during DT, such as decreased walking speed and increased step-to-step variability, have a high predictive value for fall risk and cognitive decline. Previously we showed the feasibility of DT during functional magnetic resonance imaging (fMRI) using an MRI-compatible stepping device. Here, we improved the DT-fMRI protocol with respect to task difficulty and signal robustness, making it more suitable for individualized analysis to better understand the neuronal substrates of cognitive-motor interference effects. Thirty healthy elderly subjects performed cognitive and motor single tasks (ST; stepping or finger tapping), as well as combined cognitive-motor DT during fMRI. After whole brain group level analysis, a region-of-interest (ROI) analysis and the computation of dual task costs (DTC = activation difference ratio ST/DT) at individual level were performed. Activations in the primary (M1) and secondary motor as well as in parietal and prefrontal cortex were measured at the group level during DT. Motor areas showed decreased activation whereas parietal and prefrontal areas showed increased activation in DT vs. ST. Stepping yielded more distinctive activations in DT vs. ST than finger tapping. At the individual level, the most robust activations (based on occurrence probability and signal strength) were measured in the stepping condition, in M1, supplementary motor area (SMA) and superior parietal lobule/intraparietal sulcus (SPL/IPS). The distribution of individual DTC in SPL/IPS during stepping suggested a separation of subjects in groups with high vs. low DTC. This study proposes an improved cognitive-motor DT-fMRI protocol and a standardized analysis routine of functional neuronal markers for cognitive-motor interference at the individual level.

## Introduction

### Clinical Use of Cognitive-Motor DT

Gait analysis is used as a diagnostic tool for the evaluation of fall risk and cognitive decline in older adults and is considered predictive for future development of neurodegenerative disorders such as dementia (Lundin-Olsson et al., 1997; Lindenberger et al., 2000; Li et al., 2001; Kressig et al., 2008; Herman et al., 2010; Montero-Odasso and Speechley, 2018). Spatiotemporal gait parameters can be objectively quantified using, for example, the GAITRite© electronic walkway system, which provides detailed insight into measures of gait speed or variability (Bridenbaugh and Kressig, 2011, 2014). Moreover, according to recent meta-analytic reviews (Hamacher et al., 2015; Bahureksa et al., 2017), a reliable discriminator between healthy and cognitively impaired older adults, e.g., at risk for dementia, is the measurement of gait during the simultaneous performance of a cognitive task, namely a cognitive-motor dual task (DT).

Performance decline during DT in one or both tasks is indicated by DT interference effects resulting from competing resources used by both tasks. This can be operationalized as the performance difference between the single and dual task, the so-called dual task costs (DTC). The DTC could thus index the available attentional reserve capacity, i.e., an index of how many cognitive resources are still available when performing a DT as compared to the resources used during a single task (ST). The DTC have been shown to be age dependent. Overall, decreased gait speed and increased step-to-step variability as well as larger DTC are associated with older age (Springer et al., 2006; Yogev-Seligmann et al., 2010; Holtzer et al., 2014b) and the presence of neurodegenerative disorders such as pre-stages of Alzheimer’s disease (AD), e.g., mild cognitive impairment (MCI; Bridenbaugh and Kressig, 2015; Montero-Odasso et al., 2017).

### Neural Correlates of DT

Although the concept of cognitive-motor DT is established in the clinical diagnosis, therapy and rehabilitation of neurodegenerative disorders with impairments in executive neuromotor control of gait (Bridenbaugh and Kressig, 2011, 2014, 2015), the neural correlates of DTC remain unclear. Assessing the functional neural basis of DTC in older adults might help in establishing complementary diagnostic tools to facilitate and clarify uncertain diagnoses such as the early stages of AD, i.e., mild neurocognitive disorder, in which structural brain atrophy is not yet visible.

From previous studies, we know that the cortical activity during walking is highly dependent on task complexity, age and pathologies (Menant et al., 2014; Patel et al., 2014; Hamacher et al., 2015). To this respect, brain areas such as supplementary motor areas (SMA) are relevant for gait control (Labriffe et al., 2017), while a more widespread neuronal network is active during cognitive-motor DT. This network includes: the cerebellum, precuneus, SMA, and prefrontal areas (Blumen et al., 2014; Hamacher et al., 2015), as well as temporo-parietal (Metzger et al., 2017), premotor and sensorimotor areas (Pizzamiglio et al., 2017).

More specifically, reduced activations within the inferior frontal gyrus (IFG) and occasionally in the superior parietal lobule (SPL) were identified during a cognitive-motor DT (i.e., ankle-movement and the concurrent performance of an N-back task) in young adults (Johannsen et al., 2013). Therefore, those authors proposed that the SPL and IFG might be of interest when the goal is to reduce DT interference effects such as DTC.

Studies addressing the neural correlates of DTC are methodologically heterogeneous and their results inconsistent. A recent systematic review of the neural correlates of cognitive-motor DT interference concluded mixed effects (e.g., increased or decreased activations of task-specific or task non-specific areas), hence a neural locus of DTC could not be detected, presumably due to broader network effects (Leone et al., 2017).

### DT and Aging

Additionally, although age seems to play an important role, the studies that evaluate brain activity during DT in the context of aging, are sparse and provide mixed results (Holtzer et al., 2014a). An EEG study showed, besides age-related performance differences during posture-cognition DT, increased neural oscillations in frontal, central-frontal, central, and central-parietal brain regions in elderly subjects compared to younger adults (Ozdemir et al., 2016). A functional magnetic resonance imaging (fMRI) study by Van Impe et al. (2011) showed age-related changes from ST to DT performance, i.e., elderly revealed increased fronto-parietal activation during performance of a visuo-motor task. Furthermore, both groups showed increased percent signal change of their brain activity in the (pre-) SMA during DT as compared to ST. A recent fMRI study comparing the neural correlates of cognitive-motor DT in young and old individuals demonstrated an age-related reduction in upregulation of brain activity from ST to DT (Papegaaij et al., 2017). This effect was best shown in the insula [used as a region-of-interest (ROI)] in elderly participants. Yet, this study used a balance stimulation task as a motor condition in the fMRI, and, thereby, did not specifically analyze brain activation during locomotion or gait.

Overall, due to differences in (a) *the applied neuroimaging technique and data analysis strategy* [whole brain vs. ROI, aswell as different ways of analyzing DT specific effects (Szameitat et al., 2002)] (b) *the tasks used to simulate gait* [e.g., imagined gait (Labriffe et al., 2017), anti-phase ankle dorsi-plantarflexion movements (Johannsen et al., 2013) or balance simulation (Papegaaij et al., 2017)]; but also (c) *the type of cognitive task used in the context of DT*, a direct comparison of these studies is difficult and restricts the generalization of findings. Furthermore, none of the studies described above evaluated the DT effect at the individual level. Thus, there is no consensus in terms of direction of altered activity (e.g., increased or decreased) in DT specific brain areas.

### Motivation and Study Aims

In order to evaluate the neural correlates of gait in elderly and address some of the highlighted research gaps, we developed an MRI-compatible stepping device and tested its feasibility in young and elderly adults in a previous study (Burki et al., 2017). Gait parameters measured with the GAITRite© electronic walkway system were positively correlated with the stepping parameters assessed with the stepping device, speaking in favor of the validity of the device. The results showed a general decrease of brain activation during DT as compared to ST and pointed to the SPL as a potential ROI to measure individual cognitive-motor interference effects during DT. This finding is in line with previous literature which shows that SPL activation is related to motor imagery of gait, as well as to awareness and intention of movements during DT conditions (Wagner et al., 2005; Bakker et al., 2008; Desmurget et al., 2009), but also to dual tasking and task switching performance (Johannsen et al., 2013). Therefore, SPL might play a role when attention has to be divided among different processes, e.g., during DT, and represent a target region in the evaluation of cognitive-motor interference effects (Al-Hashimi et al., 2015). Additionally, the proposed fMRI DT paradigm yielded robust cortical activations at the individual subject level, which is desirable when the goal is to assess the inter-individual variability and the future evaluation of, e.g., subjects at risk for AD.

Based on these previous results, the aims of the present study were (1) to improve the fMRI protocol developed in Burki et al. (2017) in terms of robustness of the fMRI signal to make it better suitable for individual analysis, (2) to target brain areas sensitive to cognitive-motor interference effects during DT which could be used to stratify elderly subjects (age 65 +) in different DT impairment levels (3) to evaluate if a finger tapping movement instead of stepping ensures similar results in the DT context, knowing it to be less prone to movement artifacts in the MRI.

We hypothesize that parietal and motor regions might be associated with the DT interference effects in this specific cognitive-motor DT.

Consequently, by identifying the functional neural markers of cognitive-motor DT in elderly population using a non-invasive neuroimaging technique, might facilitate and complement diagnosis and monitoring of disorders with emerging cognitive decline but where morphological changes are not evident and therefore differential diagnosis needed. Furthermore, if successful this paradigm might be used in the establishment of a complementary diagnostic imaging protocol.

## Materials and Methods

### Participants

The study sample consisted of thirty healthy elderly volunteers (mean age ± SD: 70.2 ± 4.97; mean years of education ± SD: 13.67 ± 2.76; 14 females vs. 16 males), recruited from a database for cognitively healthy volunteers at the Memory Clinic of the Department of Geriatric Medicine Felix Platter in Basel, Switzerland. Participants had no history of neurological or psychiatric disorders and reported themselves as healthy [exclusion criteria: severe sensory or motor deficits; severe auditory, visual or speech deficits; severe systemic disease; diseases with severe or probable impact on the central nervous system (e.g., neurologic disorders including significant cerebral-vascular disease, generalized atherosclerosis, and diagnosed psychiatric disorders); continuous mild-to-intense pain; and intake of potent psychoactive substances except minor tranquilizers]. Participants performed various neuropsychological tests related to executive functions, interference management and short-term working memory performance (see Supplementary Table S1).

To assure their cognitive health, participants were allowed no more than one out of normal range score (e.g., not more than one demographically adjusted *z*-score below −1.28) in the Mini-Mental State-Examination (MMSE; Folstein et al., 1975; mean score ± SD: 28.73 ± 1.08), German version of the California Verbal Learning Test (Delis et al., 1988), Trail Making Test B (Reitan, 1958), and Informant Questionnaire on Cognitive Decline in the Elderly (Jorm et al., 1989; Ehrensperger et al., 2010; see Supplementary Table S1). All participants were right-handed and the hand preference was determined by using a modified questionnaire according to Annett (1967). All subjects gave written informed consent prior to the experimental sessions. The study was approved by the local Ethical Committee Basel, Switzerland.

### Procedure

The study procedure was similar to our previous study Burki et al. (2017). Participants practiced the stepping and finger tapping task outside of the scanner. Afterward, they were positioned in the MRI scanner. The feet were fixed on the pedals of the custom-made MRI-compatible stepping device and a cylindrical cushion was placed under the knees for comfort. This stepping device allows controlled foot movements and registers the step onset time of each foot in milliseconds (ms) during scanning (Burki et al., 2017). Both hands were positioned on a button response unit, to register tap onset times during the finger-tapping task (Celeritas Fiber Optic Response System, Psychology Software Tools, United States). The stimuli of the fMRI paradigm (details described below) were projected onto a screen behind the scanner, which the participants were able to see in a mirror attached to the head coil. Movement artifacts were minimized by fixing the head with preformed foam cushions and by instructing each volunteer to gaze at a fixation point.

### FMRI Paradigm

The fMRI paradigm consisted of three different tasks: motor single task (*motorST*), cognitive single task (*cognST*), and cognitive-motor DT. During the *motorST* participants had to either step on the pedals (*stepST*) or to press the buttons alternately with their index- and middle finger of both hands at a self-selected pace (*tapST*). A symbol of the foot or hand, respectively, was presented on the screen prompting them to execute the movements. The symbol was stationary and in no way suggested a cadence. During the *cognST* participants had to perform a verbal fluency task or a serial subtraction task, i.e., naming as many words as possible from given categories (e.g., fruits, names, clothing items) or to count out loud backward as far as possible by sixes or sevens (e.g., 124 – 7, 111 – 6). The order of the two types of cognitive tasks was counterbalanced within the runs. During the DT participants had to simultaneously perform one of the *motorST* together with one of the *cognST*, i.e., either stepping and naming/counting (*stepDT*) or tapping and naming/counting (*tapDT*). While the stepping/tapping was performed in different runs, the type of *cognST* was counterbalanced within each run. The participants’ responses from the *cognST* and DT were registered by an MRI-compatible microphone (Fiber Optic Microphone for fMRI, Optoacoustics, Israel). The order of these tasks was randomized within each run.

### Experimental Design

In order to improve the robustness of the fMRI signal, we adapted the task from Burki et al. (2017) by using two cognitive tasks with varying difficulty. The fMRI paradigm was composed of seven different runs, each in a block design. Each run was composed of five blocks of 18 s baseline periods and four blocks of 36 s task periods. The block design was similar to that described in Burki et al. (2017). During the baseline blocks participants fixed their gaze on a black cross on a white screen. Task blocks consisted of one of the three task categories, i.e., *motorST, cognST*, cognitive-motor DT. Each run started and ended with a baseline block, in between the baseline and task blocks were alternated.

In the first two runs, participants had to perform one of the two *motorST*, i.e., the *stepST* or the *tapST*. In the third to the sixth run, participants had to perform the DT. Two of those four runs involved *stepDT* and the other two runs involved *tapDT*. The order of these runs was counterbalanced between participants. In run seven, participants performed the *cognST* (naming/counting). For a detailed description of the design, we refer to Supplementary Figure 1B.

### FMRI Data Acquisition and Pre-processing

High-resolution T1-weighted 3D MRI images of the brain (magnetization-prepared rapid acquisition of gradient echo sequence: repetition time 1570 ms, echo time 2.67 ms, 1 mm^3^ isotropic resolution, flip angle 9°, 192 contiguous sagittal slices, matrix size 256 mm) were acquired at 3 Tesla (Magnetom Prisma, Siemens, Erlangen, Germany) with a 20-channel head and neck coil. Additionally, blood-oxygen-level-dependent (BOLD) fMRI (echo planar imaging sequences, 38 oblique slices parallel to the anterior–posterior commissure plane, slice thickness 3 mm, gap 1 mm, repetition time 2570 ms, echo time 30 ms) were performed.

Magnetic resonance imaging images were analyzed using the BrainVoyager software (Version 2.8; Brain Innovation, Maastricht, Netherlands). Preprocessing of the data included slice scan time correction, motion correction, temporal filtering, and a voxel-wise calculation of BOLD activation using linear cross-correlations [general linear model (GLM)]. Data processing was fully standardized except for the manual overlay of functional images on structural MRI images and for the individual definition of reference points required for spatial normalization. All individual data sets were transformed to Talairach (TAL) space (Talairach and Tournoux, 1988).

### Data Analysis

#### Behavioral Data

To be able to judge the task performance during the different conditions we evaluated two different behavioral scores: during the movement tasks (stepping and tapping) we assessed movement time and variability while during the cognitive task we assessed the number of hits and errors, as further described below. These scores entered as outcome variables in the later statistical analysis which we performed using IBM SPSS Statistics 23.

##### Calculation of Behavioral Outcome Measures

From the *motorST* (*stepST*, *tapST*) we evaluated the stepping or tapping time and their variability as outcome variables for each participant. ***Stepping time*** was defined as the time elapsed between the step onset of foot 1 and step onset of foot 2 in ms. From this we calculated the *mean step time* (Sum_*steptimes*_/Number_*stepsinrun*_). The ***stepping variability*** was calculated as the coefficient of variation (CV= (standard deviation (SD)_step time_/ mean (M)_step time_ × 100). For the *tapST* we evaluated the time that elapsed between tapping with the index and middle finger for each hand. M*ean* ***tap time*** and ***tap variability*** were calculated similarly to stepping. From the *cognST*, we evaluated both the number of *correct responses* (e.g., number of correctly named words and number of correct calculations) as well as the *number of errors* per run. From the *DT*, we evaluated all dependent variables (cognitive and motor performances) for stepping and tapping, respectively.

To quantify the interference effects caused by the DT on behavioral level, we calculated the DTC for all outcome variables described above. The DTC represent the percent ratio of the difference between ST and DT performance relative to the ST performance, i.e., for the outcome variable step time we calculated the following DTC=((DT_*steptime*_-*ST*_*steptime*_)/ST_*steptime*_x100).

#### FMRI Data and Outcome Variables

##### Group level analysis

To evaluate which brain areas are active during the different tasks, we first computed group activation maps for each of the five conditions (*stepST*, *tapST*, *cognST*, *stepDT*, and *tapDT*) using a separate subjects fixed-effects (FFX) group analysis, as described in the BrainVoyager analysis pipeline^1^. In order to correct for motion artifacts, the motion correction parameters were included as confound parameters in the GLM analysis.

##### Subject level and ROI analysis

We then performed an individual analysis on the subject level using a single subject GLM analysis and by employing a dynamic threshold (see below) technique using a minimal *p* < 0.05 (FDR corrected). In order to correct for motion artifacts, the motion correction parameters were included as confound parameters in the GLM analysis. To evaluate the inter-subject variability of neural activations in our sample and stratify the subjects into different categories based on their neural responses to the different tasks, i.e., low vs. high neural DTC groups, we assessed the individual activation maps of every subject in every condition (ST and DT) in specific ROIs extracted from the previously described group analysis. The ROI selection was also based on the described DT literature and our previous work (Burki et al., 2017) as well as on their occurrence rate in individual subjects as described further down. As our participants were all right-handed, we defined all ROIs on the left-brain hemisphere. We then evaluated the occurrence of activation of every ROI in each participant.

##### Calculation of DT dependent activations

To evaluate DT specific activations, one could perform the simple contrast ST vs. DT difference. However, since our task was performed by elderly participants it needed to be designed as short as possible, to not exhaust the participants and still bring robust results on individual subject level. In addition, based on the experience from our previous work (Burki et al., 2017), we decided to use the data of the two shorter separate runs (3:59 min each), instead of using the data of one single long run (e.g., 7:35 min) for every condition (ST and DT; see Supplementary Figure 1B).

To this end, besides looking at the simple activation maps in every condition (e.g., 30 activation maps from 30 subjects during one DT run) we also analyzed the statistical parametric maps of the two DT runs combined, i.e., 30 activation maps of first DT run + 30 activation maps of second DT run = 60 activation maps. Similar we proceeded with the ST runs by adding the 30 activation maps of the pure movements (step or tap) and the 30 activation maps of the pure cognition. This had the advantage that we increased the statistical power of our analysis by having the double amount of activation maps. Furthermore, we could evaluate the effect of a combined real DT as compared to the effect of an “additive” created one (consisting of two individual STs) and evaluated by this DT specific effects. A similar analysis technique was also performed in (Szameitat et al., 2002) and was later recommended as the best but also conservative procedure to evaluate DT specific effects. These contrasts are visible in Figures 2, 3 and explained in more detailed in the result section.

##### ROI based analysis

We aimed to track DT specific ROIs, which are present in each subject and each condition. The advantage of this subject level ROI analysis is that the task dependent ROI signal could serve as discriminant factor to separate subjects with low vs. high DTC based on their individual neural response to the task.

Hence, by employing a dynamic threshold technique, individual centers of gravity (with corresponding Talairach coordinates *x, y, z*) and *t*-values for the following ROIs were determined: M1 (M1-hand = M1-H and M1-foot = M1-F), SMA, SPL/intraparietal sulcus (IPS), dorsolateral prefrontal cortex (DLPFC), and ventrolateral prefrontal cortex (VLPFC). This ROI extraction procedure has been established and used in clinical presurgical fMRI and is described in more detailed in Blatow et al. (2007) and Blatow et al. (2011).

In brief, the ROI extraction procedure was based on the following steps: (1) For each ROI the exact anatomical correlates of functional activations were assessed on transverse, sagittal, and coronal sections. (2) We used our standard cluster size of 36 mm^3^ as a spatial filter; clusters below this size were not displayed in the activation map. (3) The highest possible statistical threshold value for the correlation (*r*) between the measured BOLD signals and the applied hemodynamic reference function (HRF) was selected, (4) this threshold was gradually reduced until activations were identified in all ROIs. The lower limit of the threshold was set to *r* = 0.4 with *p* < 0.05 (FDR corrected) to ensure that BOLD signals were clearly distinguishable from background noise.

##### Statistical analysis and calculation of neural DTC

All BOLD signals were evaluated and statistically compared on an individual basis using SPSS Statistics 23. To evaluate the activation strength difference in each of the chosen ROIs, between the two conditions ST and DT in the different tasks (stepping and tapping), non-parametrical Wilcoxon Rank-Sum Tests were applied to the *t*-values measured within the ROIs.

As an equivalent to the behavioral parameter analysis and to create a counterpart of the DTC based on neural activity, we computed neural DTC using the *t-*values of the ROIs, which had the highest signal occurrence rate in subjects, i.e., M1 and SPL. The neural DTC were calculated similarly to the behavioral ones, by using the same formula (ROI *t*-value_DT_−ROI *t*-value_ST_)/ROI *t*-value_ST_× 100. The aim of this step was to have a score by which we could later separate the individuals only based on their brain activation into low vs. high DTC groups.

To assess which of the ROIs would explain most of the variance in the data and to evaluate potential effects of group (low vs. high DTC) or task (ST vs. DT) on the results, a mixed model ANOVA was conducted. Thereby the group (low DTC, high DTC), the ROI (M1, SPL), and the task (ST, DT) represented the fixed factors in the statistical model while the *t*-values of the ROIs represented the dependent variables.

The separation into low vs. high DTC was based on the distribution of DTC in the SPL ROI. The cut-off line was made based on the distribution of participants who had an occurrence of both ROIs, i.e., SPL and M1 during both ST and DT; the total sample consisted of 21 participants (see Figure 5C for the cut-off line).

## Results

The analysis of motion parameters showed that none of the subjects exhibited movements larger than > 5 mm translation or > 1° rotation during the *tapST*, *cognST*, and *tapDT* fMRI tasks. With exception of two volunteers in *tapDT* and one volunteer in *cognST*, the motion parameters were smaller than 3 mm. During the stepping tasks, two participants showed movements’ lager than 5 mm in *stepST* and six in *stepDT.* These participants were still included in the analysis after careful examination of their individual data.

### Task Dependent Group Level fMRI Activations in Single and Dual Tasks

**FIGURE 1:**
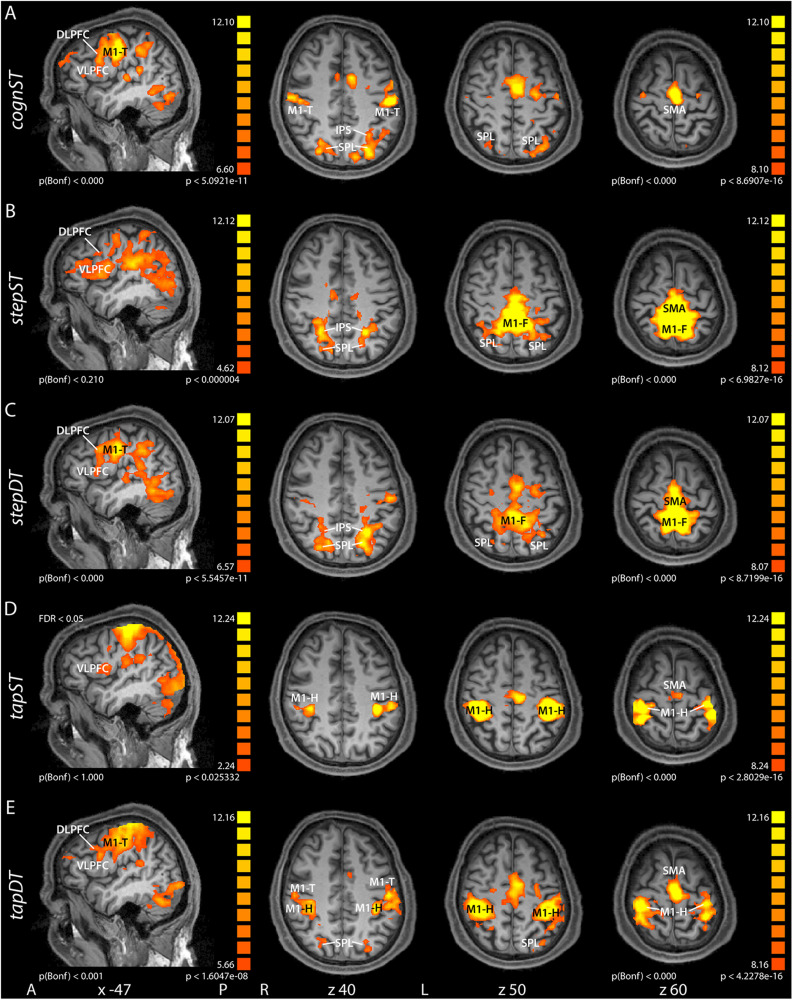
Group contrast activation maps of task versus baseline for the different conditions: motor single task (ST) and cognitive-motor dual task (DT) for stepping (step - **B**) or tapping (tap - **D**), respectively, as well as for the cognitive single task (*cognST* - **A**). In the DT conditions, we used two runs for stepping **(C)** and tapping **(E)**, respectively. This figure illustrates the results from the second run. Group activation maps were rendered onto sagittal and transversal brain slices of one participant. The results of the different runs are presented at different threshold levels. There is also a threshold difference between some transversal and sagittal planes. The threshold was chosen such that the relevant activations can be seen on the group maps. The transversal sections and the two sagittal sections *cognST* and *stepDT* were all corrected using the same threshold. All the other sagittal sections (*stepST*, *tapST*, and *tapDT*) were corrected at different thresholds (see values in image). Abbreviations: A, anterior; P, posterior; L, left; R, right; *x*, *z*, Talairach coordinates; M1, primary motor cortex (F, foot; T, tongue; H, hand); SMA, supplementary motor area; SPL, superior parietal lobe; IPS, intraparietal sulcus; VLPFC, ventrolateral prefrontal cortex; DLPFC, dorsolateral prefrontal cortex.

In the *cognST* condition, the most significant group level activations when computing the contrast task vs. baseline were measured in frontal, primary and secondary motor, and parietal areas (Figure 1A), more specifically in DLPFC, VLPFC, SMA, SPL, IPS, and M1 within the presumable representation of the tongue (M1-T). During the *motorST*s (*stepST* or *tapST*), the most significant group level activations were measured in (Figures 1B,D): the secondary motor cortex, in particular SMA, the primary motor cortex within the respective representations, i.e., foot motor area (M1-F) in *stepST* and hand motor area (M1-H) in *tapST* and left lateralized VLPFC. Additionally, during *stepST* clusters in parietal areas [SPL, inferior parietal lobule (IPL) and parts of the IPS] as well as in DLPFC showed higher significance as compared to *tapST*. During the cognitive-motor DT (*stepDT* and *tapDT*), co-activation of the aforementioned areas could be observed (Figures 1C,E), i.e., in the prefrontal (DLPFC, VLPFC) and primary motor areas, depending on which motor task was performed (M1-H in *tapDT* or M1-F in *stepDT*) and M1-T in both DTs as well as SMA and parietal areas (SPL, IPL, and IPS).

**FIGURE 2:**
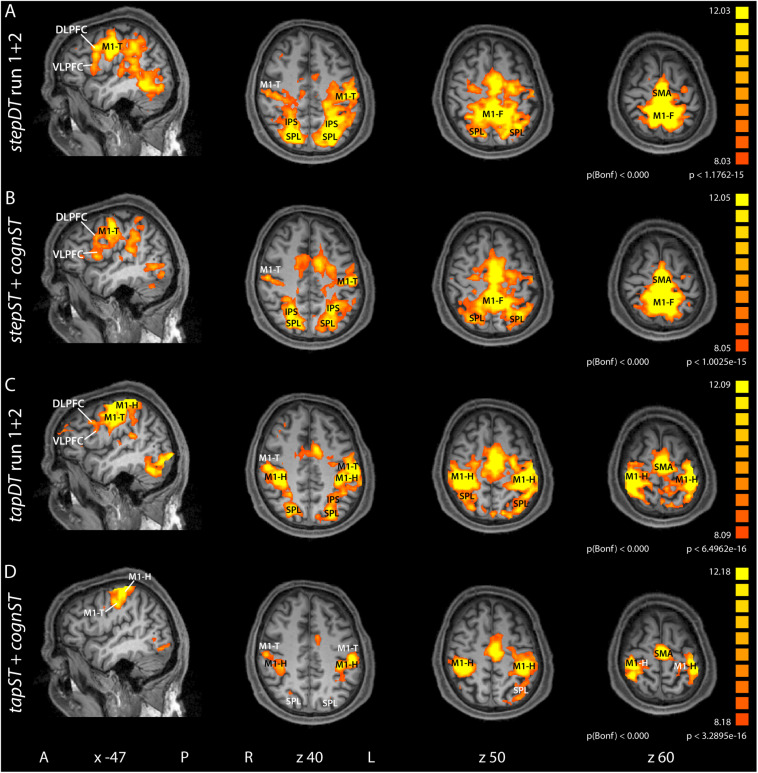
Group contrast activation maps of task versus baseline for the dual task (DT) conditions and the additive single task (ST) conditions for stepping and tapping, respectively. The *stepDT* run 1 + 2 **(A)** and the *tapDT* run 1 + 2 **(C)** contrasts contain the activation maps from both DT runs, for stepping and tapping, respectively. The contrast *stepST* + *cognST* **(B)** contains the activation maps from the *stepST* run and the *cognST* run. Similarly, the contrast *tapST* + *cognST* **(D)** contains the activation maps from the *tapST* run and from *cognST* run. Hence, the contrasts contain 60 activation maps each (30 participants × 2 runs). Group activation maps are rendered onto sagittal and transversal brain slices of one participant. Abbreviations: A, anterior; P, posterior; L, left; R, right; *x, z*, Talairach coordinates; M1, primary motor cortex (F, foot; T, tongue; H, hand); SMA, supplementary motor area; SPL, superior parietal lobe; IPS, intraparietal sulcus; VLPFC, ventrolateral prefrontal cortex; DLPFC, dorsolateral prefrontal cortex.

In view of the large overlap of involved brain areas in ST and DT, we wanted to evaluate DT-specific BOLD activations. For this reason, we investigated the relative contribution of the involved brain areas to the processing of ST vs. DT. Thereby, activation maps obtained during both runs of DT were compared to the additive activation maps of the two corresponding STs. To balance the amount of data in this comparative analysis, statistical parametric maps were computed as follows: *stepDT* run 1 + 2 (Figure 2A and Table 2); *stepST* + *cognST* (Figure 2B and Table 2); *tapDT* run 1 + 2 (Figure 2C and Table 2); *tapST* + *cognST* (Figure 2D and Table 2) and as described in section “FMRI Data and Outcome Variables” *Calculation of DT dependent activations*.

**FIGURE 3:**
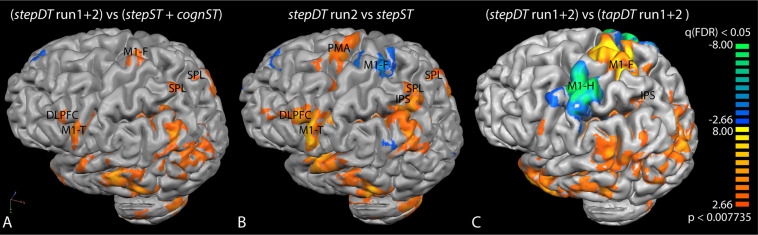
Group activation maps of the contrasts **(A)** *stepDT* (run1 + 2) vs. additive ST runs (*stepST* and *cognST*), **(B)** *stepDT* run 2 vs. *stepST*, and **(C)** *stepDT* (run1 + 2) vs. *tapDT* (run1 + 2). Functional magnetic resonance imaging (fMRI) activations were rendered onto a brain surface and the left hemisphere is shown. The activation threshold was set at FDR < 0.05. The color scale indicates the following: orange/yellow clusters represent increased activation in the indicated contrast and blue/green clusters represent the areas which were active in the opposite contrast. Abbreviations: M1, primary motor cortex (F, foot; T, tongue; H, hand); PMA, pre-motor area; SPL, superior parietal lobe; IPS, intraparietal sulcus; DLPFC, dorsolateral prefrontal cortex.

When computing the contrast of *stepDT* (two runs) vs. *stepST* + *cognST* conditions at the group level, significantly increased activations in the primary motor areas (M1-F, M1-T) as well as in SPL and DLPFC could be found (Figure 3A and Table 1). The group level results of the contrast *DT* vs. *ST* of the stepping condition revealed significantly increased activations in *stepDT* as compared to *stepST* in the secondary motor areas (SMA and PMA), M1-T, DLPFC, and SPL. In the opposite contrast *stepST* vs. *stepDT*, an increased activation in M1-F was observed (Figure 3B and Table 1).

**TABLE 1:** Talairach (TAL *x, y, z*) coordinates of the centers of gravity of the active areas shown in the group contrast activation maps in Figure 3. Additionally, the *t-*value, *p*-value, and the number of voxels in the respective active cluster are presented (FDR corrected, *p* < 0.05). Clusters in bold represent the significant clusters (blue/green) from Figure 3.

**fMRI Map**	**ROI**	**TAL *x***	**TAL *y***	**TAL *z***	***t-*value**	***p*-value**	**Number of Voxels**
(*stepDT* run 1 + 2) vs (***stepST* + *cognST***)	M1-F	−8	−40	56	4,0	0,000113	109
	M1-T l	−46	−16	31	5,4	0,000000	218
	M1-T r	49	−14	30	5,0	0,000001	123
	SPL r	31	−71	29	4,6	0,000013	171
	SPL l	−26	−63	28	5,4	0,000000	178
	DLPFC l	−43	2	24	3,6	0,000684	183
*stepDT* run 2 vs ***stepST***	**M1-F**	**−2**	**−33**	**59**	**4,9**	**0,000002**	**258**
	M1-T l	46	−14	30	6,5	0,000000	224
	M1-T r	−48	−15	29	8,5	0,000000	210
	SPL r	32	−67	40	5,1	0,000002	244
	SPL l	−28	−66	31	5,9	0,000000	303
	PMA r	27	−8	55	4,5	0,000019	348
	PMA l	−24	−4	50	4,7	0,000003	115
	DLPFC l	−42	−2	33	5,9	0,000000	193
(*stepDT* run 1 + 2) vs (***tapDT* run 1 + 2**)	M1-F	0	−34	57	12,5	0,000000	146
	**M1-H l**	**40**	**−26**	**51**	**11,4**	**0,000000**	**218**
	**M1-H r**	**−44**	**−26**	**53**	**9,8**	**0,000000**	**165**
	SPL r	19	−66	27	4,2	0,000040	328
	SPL l	−17	−66	35	3,6	0,000373	134

*M1, primary motor cortex (F, foot; T, tongue; H, hand); PMA, pre-motor area; SPL, superior parietal lobe; DLPFC, dorsolateral prefrontal cortex; l, left; r, right; *stepST*, stepping single task; *stepDT*, stepping dual task; *tapDT*, tapping dual task; *cognST*, cognitive single task; fMRI, functional magnetic resonance imaging.*

The group activation contrast *stepDT* (run1 + 2) vs. *tapDT* (run1 + 2) showed increased activations in M1-F and parietal regions (SPL/IPS). The opposite contrast *tapDT* (run1 + 2) vs. *stepDT* (run1 + 2) showed increased activations in M1-H (Figure 3C and Table 1).

### Task Dependent ROI Based Activations on Subject Level

**FIGURE 4:**
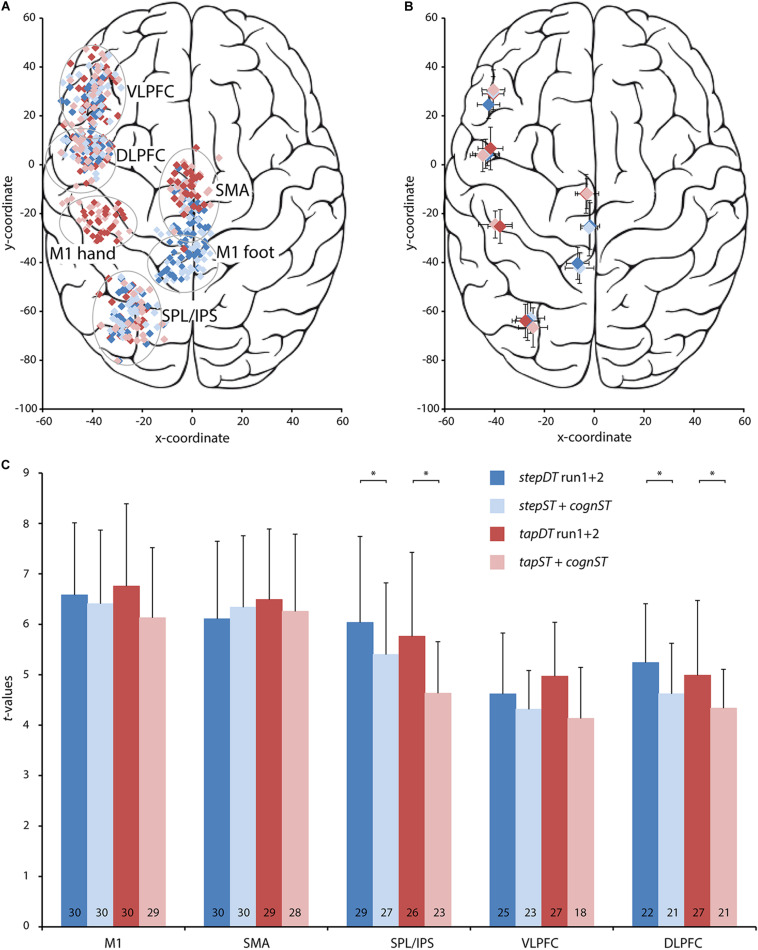
Spatial coordinates and *t-*values at the centers of gravity of individual activations for five regions of interest (ROI) in each one of the four conditions, i.e., the dual task (DT) conditions and the additive single task (ST) conditions for stepping (blue color), and tapping (red color), respectively. These results are based on the data from the same contrasts as in Figure 2 and Table 2. All analyses were performed in the left hemisphere. Darker colors represent the two DT conditions (*stepDT* run 1 and 2, *tapDT* run 1 and 2) and lighter colors the additive ST conditions (stepST + *cognST*, *tapST* + *cognST*) for stepping and tapping, respectively. **(A)** Plotted are the Talairach coordinates (*x*, *y*) of the centers of gravity of activation in each of the five ROIs for each individual subject. Conditions are color-coded as depicted in the legend. **(B)** Plotted are the Talairach coordinates (*x, y*) of the mean center of gravity over all subjects for each of the five ROIs in each of the color-coded conditions. **(C)** Plots of the mean *t-*values and their standard deviations (SD) for each ROI and each condition. The values at the bottom of each bar indicate the number of subjects in which functional magnetic resonance imaging (fMRI) activation in the specific ROI occurred per condition, the maximum value is 30. Abbreviations: M1, primary motor cortex; SMA, supplementary motor area; SPL/IPS, superior parietal lobe/intraparietal sulcus; VLPFC, ventrolateral prefrontal cortex; DLPFC, dorsolateral prefrontal cortex; ^∗^, significant difference, *p* < 0.05.

To assess the significance of these results on a subject level, ROI based analysis was performed on the individual contrast maps for DT (*stepDT* run1 + 2 *or tapDT* run1 + 2) and the additive ST (*stepST* + *cognST or tapST* + *cognST*) conditions in each subject. Based on the previously reported group activation maps, the following ROIs were chosen and defined in the left hemisphere: M1-F, M1-H, SMA, SPL/IPS, VLPFC, and DLPFC. The spatial coordinates of the individual BOLD activations were largely overlapping between DT and ST conditions (Figures 4A,B). The *t*-values of BOLD activations in M1 and SMA did not significantly differ between DT and additive ST conditions. However, *t*-values in SPL/IPS were significantly higher in both *stepDT* and *tapDT* as compared to the respective additive ST (tap: *p* = 0.013; step: *p* = 0.032; Figure 4C). Similarly, in prefrontal areas, *t*-values were higher in DT as compared to additive ST conditions; however, this difference was only significant for DLPFC (tap: *p* = 0.036, step: *p* = 0.031; Figure 4C). The occurrence probability of activation in the different ROIs was highest in M1-F and M1-H (> 99%) as well as SMA (> 97%) in all tasks. The SPL/IPS activation occurrence probability was higher in stepping (90%) as compared to the finger tapping task (82%) and lower in VLPFC (77%) and DLPFC (76%), in particular in the additive ST conditions (Figure 4C).

### Neural Correlates of Dual Task Costs

**FIGURE 5:**
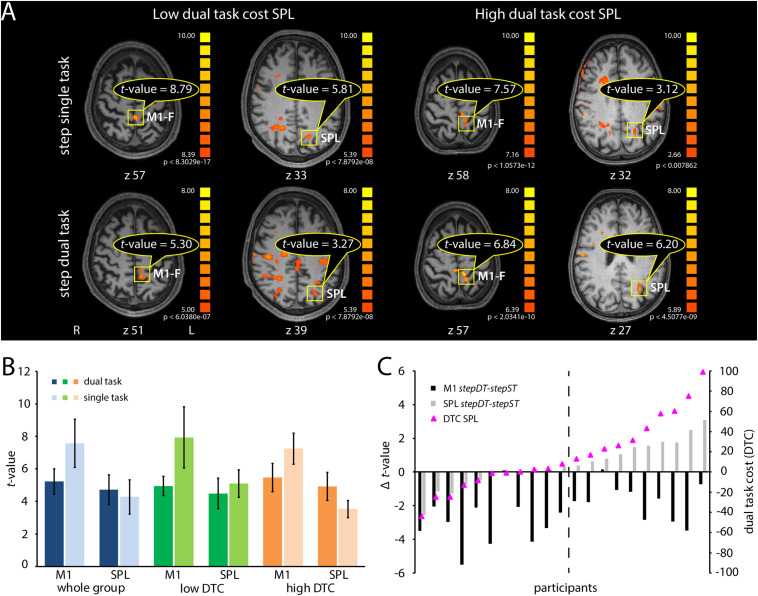
Neural representation of dual task costs (DTC) and *t-*values in M1 and SPL, in the stepping single task (ST), and dual task (DT) conditions after individual analysis using a dynamic thresholding technique. **(A)** Descriptive visualization of *t-*values in M1 and SPL in two subjects, one with low SPL DTC (first two columns) and one with high SPL DTC (last two columns) in the stepping conditions. The DTC in SPL are calculated as the (SPL *t-*value _DT_ – SPL *t-*value _ST_)/SPL *t-*value _ST_ × 100. The first row represents the activation in *stepST* and the second row in the second run of the *stepDT*. The *t-*values of M1 and SPL activations are lower in DT than in the ST in the subject with low DTC in SPL. In the subject with high DTC in SPL, the *t-*value in M1 is smaller in DT compared to ST whereas in SPL it is the opposite, i.e., *t-*value is higher in DT than in ST. **(B)** Plot of the group-based mean *t-*values with standard deviations of the M1 and SPL activation for ST (lighter colors) and DT (darker colors) stepping conditions. Blue colors refer to the whole group (21 subjects), green colors to the group with low DTC in SPL and orange to those with high DTC in SPL. **(C)** Black bars represent the *t-*value difference between DT and ST in M1 in each subject. Gray bars indicate the difference between DT and ST in SPL in each subject. Pink triangles represent the DTC values in SPL for each subject. This plot includes 21 subjects, which had activations in both regions of interest (M1 and SPL) in both conditions (ST and DT). The difference in *t-*value is calculated as “*t-*value DT - *t-*value ST” for each region of interest. The cut-off line represents a descriptive visualization of the separation between the groups with low DTC vs. the group with high DTC. By DT, we refer to the activation maps of the second DT run (*stepDT*), by ST we refer to the activation maps of the single motor task run (*stepST*). Abbreviations: M1-F, primary motor cortex foot; SPL, superior parietal lobe; L, left; R, right; z, Talairach coordinates; *stepST*, stepping single task; *stepDT*, second run of stepping dual task.

Based on the obtained results, we proceeded with the stepping paradigm for further individual investigation. M1-F and SPL/IPS were chosen as target ROIs considering the high occurrence probability of activation in individual subjects and the distinct activation changes in these ROIs between ST and DT (Figure 4C). As a minimal requirement for an informative and robust result on individual level, we included one run *stepST* and one run *stepDT* in the individual ROI analysis (Figure 5A). Twenty-one subjects could be evaluated, showing activation in both tasks and both ROIs (Figure 5B, blue bars, whole group). To quantify activation differences between ST and DT, we calculated the DTC in SPL/IPS and M1-F in each individual subject. *T*-values in M1-F were decreased on average and, in most individuals, in *stepDT* as compared to *stepST* (Figure 5B, blue bars; Figure 5C, black bars). In contrast, *t*-values in SPL/IPS were, on average, comparable in *stepDT* as compared to *stepST* (Figure 5B, blue bars). However, when analyzing the distribution of the individual activation differences between ST and DT in SPL/IPS, two groups could be distinguished in terms of DTC in SPL/IPS, i.e., one group with increasing SPL/IPS activation from ST to DT (high DTC) and one group with decreasing SPL/IPS activation from ST to DT (low DTC; Figure 5B, green and orange bars and Figure 5C, pink triangles).

The ANOVA results showed stronger activation of M1-F than SPL/IPS [main effect of ROI: *F*(1,19) = 33.29, *p* = 0.001)]. The *t*-values were higher during ST than during DT conditions [main effect of task *F*(1,19) = 82,57, *p* < 0.001]. Furthermore, the analysis revealed two interaction effects. First, a task × ROI interaction effect [*F*(1,19) = 40.35, *p* < 0.001], suggesting that the activation difference between ST and DT is influenced by the two different ROIs. Second, we found a ROI × group interaction effect [*F*(1,19) = 21.52, *p* < 0.001], suggesting that the activation difference between the two ROIs is also influenced by the DTC differences (low vs. high).

### Behavioral Dual Task Costs

The behavioral data obtained during fMRI, i.e., stepping, tapping parameters and cognitive performance, go in line with the SPL DTC distribution in these low or high DTC groups. More specifically, the low DTC group showed lower step time, lower step variability, better cognitive performance and fewer errors than the high DTC group. However, these differences were only trends and not statistically significant (Supplementary Figure S2).

## Discussion

In this study we fulfilled three main aims: (1) we improved the previously developed fMRI protocol (Burki et al., 2017); (2) we tracked DT specific brain areas to separate elderly groups based on their performance; (3) we showed that stepping yields more robust results than finger tapping and therefore should be chosen as the motor counterpart within the DT. Based on the obtained results, we conclude that a motor ST and a cognitive-motor DT run are the minimal requirements for the fMRI protocol to detect robust activity on the individual level in healthy older adults, which fulfills clinical application criteria.

### Stepping vs. Finger Tapping in a Cognitive-Motor Dual Task

Although DT and ST performance using tapping has been evaluated in previous studies (Wu et al., 2013; Soylu and Newman, 2016; Crockett et al., 2019) the question which motor system is the most reliable and useful in evaluating the neural correlates of DTC under MRI conditions has not been evaluated until now. Present results showed that, while the behavioral parameters of stepping and tapping were comparable, a significant difference could be observed in the neural correlates, i.e., tapping yielded a generally lower signal occurrence rate in individual subjects and produced an overall lower signal intensity in DT specific ROIs (*t*-values), i.e., in SPL/IPS and PFCs (Figures 3C, 4 and Table 1). Hence, the lower signal occurrence rate and intensity in this specific region during the tapping condition limits the evaluation of DT interference effects on single-subject level.

Overall, the results suggest that moving the hand – and performing a cognitive task – might be a more automatized process, than doing the same task with the foot, resulting in lower brain activity. As discussed in previous studies by Poldrack et al. (2005) and Leone et al. (2017) the amount to which multitasking is demanding depends on the level of automatization and training and whether the performed tasks involve identical brain areas. Considering this and accounting the results in our previous study (Burki et al., 2017), we speculate that the tapping DT condition might be less demanding to evoke DT specific activation in SPL/IPS and, therefore, the stepping condition may be the more appropriate task to evaluate DT performance and interference effects such as DTC. Furthermore, functionality of the lower extremity is of greater clinical importance in old age, as it is associated with mobility, gait safety, and fall risk (Kressig et al., 2008; Herman et al., 2010; Bridenbaugh and Kressig, 2011, 2014).

### The Neural Network of DT

#### Motor Areas

The activation of M1 and SMA during DT was expected, as it is known that – amongst others – these areas are responsible for planning, control, and execution of movements. These areas also yielded the highest occurrence rate of activation on subject level in the present study. M1 and SMA have been shown to be equally activated in real as well as in imagined gait (Miyai et al., 2001; La Fougere et al., 2010) and to exhibit a clear role distribution in gait initiation (primary motor areas) and gait control (SMA; Leisman et al., 2016). Hence, although the gait like movement was performed in a supine position, it elicited activation of the motor neural network, speaking in favor of the appropriateness of this task and its gait-similar function in the MRI context.

#### Prefrontal Areas

In the present study, during the cognitive-motor DT, we found left-lateralized co-activation of the DLPFC and VLPFC, which are known to be associated to executive functions such as working memory, cognitive flexibility and inhibition (Miller and Cummings, 2007). These activations can be interpreted as neural responses to the cognitive part of the cognitive-motor DT in which participants had to either perform a subtraction task or enumerate words of certain categories. However, the activation of these frontal brain regions in a DT context was not consistently found, possibly because this effect is rather age- and task complexity-dependent (Cabeza, 2001; Beurskens et al., 2014). Findings go also in line with a study by (Szameitat et al., 2002), which found also a certain specificity of left inferior frontal sulcus when participants managed the interfering information of two choice reaction tasks in the fMRI, the difference being that the DT didn’t contain any motor action.

Furthermore, present findings support the proposed hypothesis that aging results in an adaptation of brain activation (Davis et al., 2008) with a more intense recruitment of anterior compared to posterior brain areas. Thereby, increasing frontal activation during healthy aging has been interpreted as a reorganization of brain function due to the difficulty of effectively dividing neural resources during DT (Szameitat et al., 2006). However, although present results show frontal activations in elderly during both ST and DT, with higher activations during DT, the direction of activation change (increased or decreased) and its contribution to these brain reorganization mechanisms remain unclear.

Furthermore, these functional compensation mechanisms might be linked to the natural occurrence of brain atrophy during lifetime. Previous studies showed that PFC atrophies more than other regions (Dumurgier et al., 2012; Storsve et al., 2014; Doi et al., 2017) and is also often functionally underutilized with age (Holtzer et al., 2011). Several studies showed an association of DT performance (i.e., processing speed and executive function) and the gray matter volume of various areas of motor control including PFC (Allali et al., 2019; Blumen et al., 2019; Tripathi et al., 2019). Albeit structure-function evaluations of these regions are scarce, first evidence indicates the potential moderator role, which brain prefrontal activation can have on volume loss in elderly (Wagshul et al., 2019).

#### Parietal Areas

Group level results revealed an activation of parietal areas (SPL/IPS), which similar to prefrontal areas, was more pronounced in the DT as compared to the additive ST condition (see Figure 2 and Table 2 for group results and Figure 3 and Table 1 for group contrast results). These results highlight the presence of a complex network of interacting brain areas involved in DT interference effects.

**TABLE 2:** Individual Talairach (TAL *x, y, z*) coordinates and mean *t-*values (± SD) of the centers of gravity (COG) of activation in each condition. These COGs are based on the activation maxima of the contrast task vs. baseline during the respective conditions. Additionally, the number of voxels in each cluster and the occurrence rate (number of subjects) of activation per region of interest (ROI) are indicated.

**fMRI Task**	**ROI**	**TAL *x***	**TAL *y***	**TAL *z***	***t*-value**	**Number of Voxels**	***n* ROI**
*stepDT* run 1 + 2	M1	−70.8	−401.2	580.5	6.60.3	21126	30
	SMA	−20.7	−251.7	600.7	6.10.3	20417	30
	SPL	−270.9	−641.5	301.2	6.00.3	27244	29
	VLPFC	−420.9	251.1	271.0	4.60.2	20926	25
	DLPFC	−431.0	40.9	300.9	5.20.2	21417	22
*tapDT* run 1 + 2	M1	−380.9	−251.3	531.5	6.80.3	20719	30
	SMA	−30.6	−121.5	601.0	6.50.3	18515	29
	SPL	−281.0	−641.3	301.5	5.80.3	23230	26
	VLPFC	−410.8	291.4	271.3	5.00.2	16614	27
	DLPFC	−421.0	71.7	311.3	5.00.3	17810	27
*stepST* + *cognST*	M1	−61.1	−421.2	600.6	6.40.3	24122	30
	SMA	−20.6	−262.1	600.6	6.30.3	20918	30
	SPL	−261.1	−631.2	321.3	5.40.3	22418	27
	VLPFC	−401.2	301.6	251.3	4.30.2	19318	23
	DLPFC	−441.1	51.3	311.3	4.60.2	19321	21
*tapST* + *cognST*	M1	−401.3	−241.0	491.2	6.10.3	20518	29
	SMA	−30.9	−121.2	580.8	6.30.3	17513	28
	SPL	−251.2	−671.7	320.9	4.60.2	24132	23
	VLPFC	−401.1	311.9	231.0	4.10.2	20425	18
	DLPFC	−451.1	41.4	311.1	4.30.2	18818	21
*stepDT* run 2	M1	−41.2	−391.1	580.6	5.10.2	30238	29
	SMA	−10.8	−202.2	600.7	4.90.2	26634	27
	SPL	−271.0	−641.0	301.2	4.80.2	27044	25
*stepST*	M1	−60.8	−411.0	590.3	7.40.3	20417	30
	SMA	−40.7	−291.4	590.5	7.00.3	20820	28
	SPL	−261.4	−631.7	293.0	4.30.2	21725	23

*M1, primary motor cortex; SMA, supplementary motor area; SPL, superior parietal lobe; VLPFC, ventrolateral prefrontal cortex; DLPFC, dorsolateral prefrontal cortex; *stepST*, stepping single task; *stepDT*, stepping dual task; *tapST*, tapping single task; *tapDT*, tapping dual task; *cognST*, cognitive single task; fMRI, functional magnetic resonance imaging.*

Present findings suggest that the simultaneous performance of two tasks yields not simply the additive signal of the single tasks, but rather a more complex brain pattern. This is in line with the results by Szameitat et al. (2002), who used a similar analysis approach to evaluate the DT related network and suggested that although conservative this approach is recommended if the goal is to obtain DT specific effects. The authors also compared the dual task situation to the summed activation of the single component tasks, and found that DLPFC and superior parietal regions (IPS) are involved in the coordination of concurrent and interfering task processing.

The function of these parietal areas can vary depending on the parietal subpart, e.g., the posterior part is strongly related to task switching, while the border between the anterior and posterior part (IPS) is linked to attention and higher order control of action (Tunik et al., 2007). Furthermore, it has been shown that the lateral IPS operates as an interface of perception, action, and cognition (Gottlieb, 2007) by specifying attentional priority as a synthesis of multiple task demands.

### The Neural Correlates of DTC

In the present study we made the first attempt to create a clinically useful neural marker for DTC based on a simple cognitive-motor DT in the fMRI. For this, the robust activation of DT specific areas at the individual subject level is a mandatory prerequisite. Individual level results showed that besides the high occurrence rate of activation in motor areas in every participant, the next highly occurring brain region during DT was SPL/IPS (Figure 4). The improvement in comparison to the design in Burki et al. (2017) was quantifiable, e.g., the occurrence rate of especially the SPL in the *stepDT* could be raised from 60 to 90%. On average, SPL showed a significant difference in activation strength between ST and DT. This significant difference in activation strength (presumed DT specificity) and high occurrence probability in most individuals motivated the calculation of DTC in SPL/IPS as a neural pendant to the clinically used behavioral DTC.

This computation helped in descriptively dividing the healthy elderly in two groups, one with low vs. one with high DTC (see Figure 5C). This finding is promising, as it suggests a certain specificity of this region in assessing DTC on brain functional level even in healthy non-impaired elderly subjects. Thinking further, if SPL/IPS proved to show robust DT specificity in larger clinical samples, it could later serve as reference region to assess and compare activity between healthy elderly and cognitively impaired subjects, e.g., MCI patients, on the individual level.

The idea of task prioritization at the cost of performance reduction in one of the tasks, is observable when only monitoring behavior (Pashler, 1994). A similar principle of resource use might be conceivable at the brain functional level during DT. Thus, when the neural capacities are shared, a compensation mechanism is enabled that either requires activation of additional areas (Szameitat et al., 2002), or the involved network shows a change in the mean activation, reflected in over- or under-additive activation effects during DT conditions (Just et al., 2001). Age-related reduction in up-regulation of brain activity from ST to DT has been also shown by other studies (Papegaaij et al., 2017), although the comparability is limited due to methodological differences in the performed task and non-overlapping brain areas.

Another explanation for a much broader implicated network in DT was also discussed in Tripathi et al. (2019). The authors hypothesize that structural brain loss in regions involved in DT, might contribute to an impaired neural functioning during DT and consequently prompt a compensation by alternative pathways. The exact network and the pattern of activation needs to be further investigated, e.g., evaluating the direction of activation change during task and its association to the structural atrophy.

Conclusively, the representation of DTC in brain activity is not clear yet, some tasks result in increased, some in decreased and some in no activation change during DT in the brain. These divergences might also be related to – besides the mentioned methodological and design issues – the analysis strategy of DT experiments. In our study, we used the most conservative option as suggested by Szameitat et al. (2011). By comparing the signal from the DT conditions to the sum of the STs, we aimed to disentangle the brain regions that are DT specific. Like discussed in more detail in Szameitat et al. (2011), this way of analyzing DT specific effects can elucidate brain regions or activation changes, which could not be detected when looking at the single or dual tasks alone. Furthermore, in the present study we evaluated DT specific effects also on individual level, as opposed to most previous works. This allowed us to assess interindividual differences, which are underestimated at group level. See also Figure 4, i.e., the DT specific activation in relevant areas seen on group level is not always detectable on the individual level in every subject.

### Do Neural DTC Reflect the Behavioral Performance?

The obtained pattern of DTC in SPL/IPS was in line with the observed pattern of DTC of behavioral and neuropsychological performance scores during DT in the fMRI. The group with low neural DTC in SPL/IPS had also reduced step time, reduced step variability, increased cognitive performance, fewer errors, and lower cognitive interference scores in neuropsychological tests than the group with high DTC. Most probably due to the small sample size and the fact that all participants were cognitively healthy, these differences in the behavioral parameters, as well as the associations of neural DTC in SPL and performance-based DTC showed only trends (Supplementary Figure S2). Yet, the trends in behavioral score differences confirm the results from previous studies, demonstrating that older adults rely on more cognitive resources for procedural memory tasks, e.g., during walking. Therefore, low performers show a higher motor dual task interference than high performers (Lindenberger et al., 2000; Burki, 2016).

### Limitations

A major limitation of this pilot study is the moderate sample size, as compared to the usually large study populations of behavioral neuropsychological studies. A further limitation is the intrinsically high inter- and intra-individual variability and susceptibility of the fMRI signal, making every attempt of individual measure challenging. The use of a cross-sectional design limits the causal interpretability of the data. The generalizability of the present results is restricted to cognitively healthy individuals. Furthermore, the data is influenced by the natural occurring brain atrophy with age and its interindividual variability in addition to vascular risk factors.

## Conclusion

This study presents an improved fMRI protocol for measuring the neural correlates of cognitive-motor DTC at the individual level in the elderly, eliciting robust fMRI activation. Our protocol has good applicability in healthy elderly subjects and does not require much pre-training. The novel results indicate upregulation differences in ST and DT in a network containing, among others, SPL/IPS, PFC, and M1. To confirm the significance of the observed neural correlates, future studies are needed to apply the protocol in a larger sample of healthy elderly and to include participants with varying degrees of cognitive impairment.

## Data Availability Statement

The raw data supporting the conclusions of this article will be made available by the authors, without undue reservation.

## Ethics Statement

The studies involving human participants were reviewed and approved by Ethikkommission Nordwest- und Zentralschweiz (EKNZ) Hebelstrasse 53 4056 Basel. The patients/participants provided their written informed consent to participate in this study.

## Author Contributions

JR, OR-O, and CB performed the data analysis and together with MB did the data interpretation. JR and OR-O were drafting the manuscript and OR-O performed the main literature research. CB, SK, and SB acquired the data. CB, RK, and MB designed the study. RK, CS, and MB supervised the study and edited and reviewed the manuscript. All authors revised the manuscript critically for important intellectual content.

## Conflict of Interest

The authors declare that the research was conducted in the absence of any commercial or financial relationships that could be construed as a potential conflict of interest.

The handling editor declared a past co-authorship with one of the authors LM.

## Footnotes

1 https://brainvoyager.com/bv/doc/UsersGuide/StatisticalAnalysis/FixedEffectsAnalysis/FixedEffectsGroupAnalysis.html
