# Supplementary material for: Neural Correlates of Stepping in Healthy Elderly: Parietal and Prefrontal Cortex Activation Reflects Cognitive-Motor Interference Effects
Source: Front Hum Neurosci. 2020 Sep 29;14:566735. doi: 10.3389/fnhum.2020.566735 (PMC7550687; doi:10.3389/fnhum.2020.566735)
Supplement: Supplementary file 1 [file Table_1.DOCX]

Supplementary Material

# Supplementary Data

# Supplementary Figures and Tables

## Supplementary Figures

**
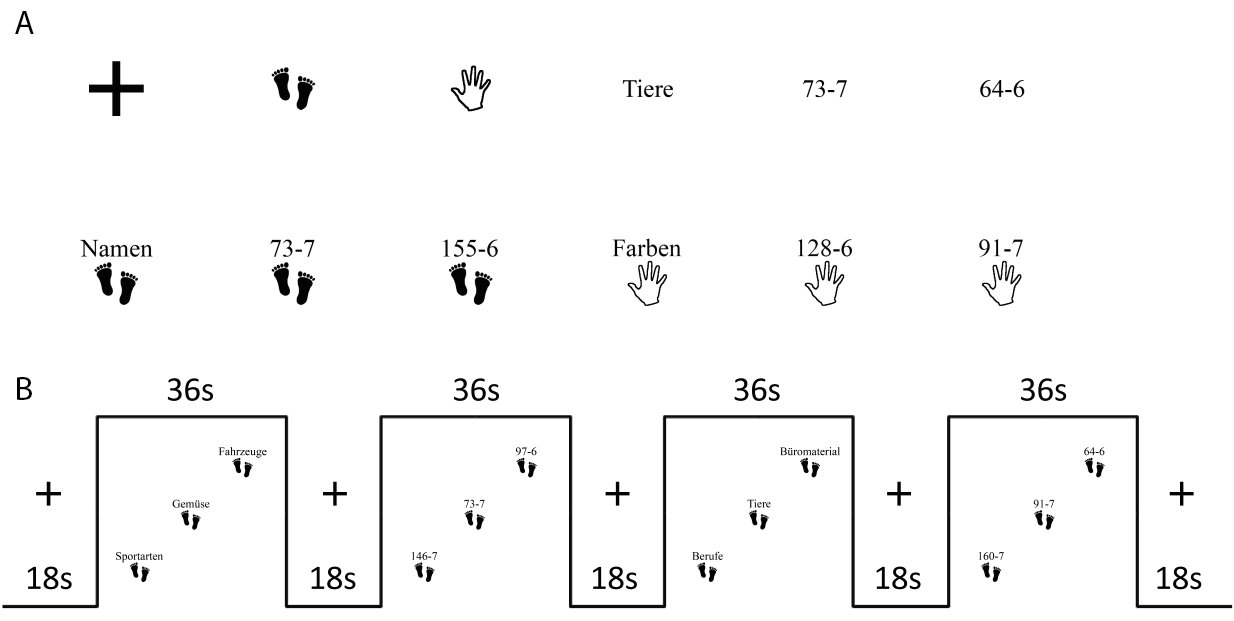
**

**Supplementary Figure 1.** Stimuli of fMRI task (A), final version of optimized fMRI task (B). Symbol specification: “+”- resting/baseline periods; feet symbol - stepping period; hand symbol - tapping period; “Tiere”(German for animals) - example for the verbal fluency task in which participants had to enumerate words from various categories; “73-7/64-6 etc.” - example for the serial subtraction task in which participants had to perform and speak out loud serial subtraction from a certain number by 6 or by 7 (counterbalanced during one run); “18s”- duration of baseline blocks; “36s”- duration of task blocks. Symbols shown together (e.g. feet symbol & 73-7) indicate that a dual task needed to be performed.


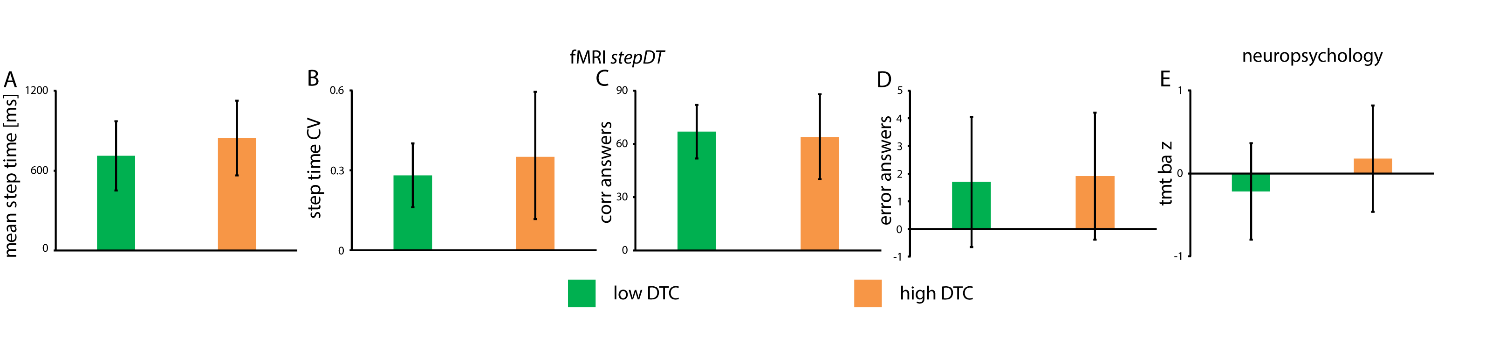
**Supplementary Figure 2.** Mean behavioral scores with standard deviations during fMRI and neuropsychological testing. Green bars represent the average score in the group with low dual task costs (DTC) in SPL, while orange bars represent the high DTC group. The cut-off line is depicted in Figure 5C.

A-D) Stepping parameters as obtained during fMRI task. x- axis representation of the two groups, y-axis average values of A: step time in milliseconds (ms), B: step time variability calculated as coefficient of variation (CV=standard deviation (SD) step time / mean (M) step time), C: amount of correct responses in cognitive task during DT, D: amount of errors in cognitive task during DT

E) Average task switching cost values in the neuropsychological Trial Making Test (TMT)

| Task | Cognitive performance | Dependent variable | Mean (SD) | Reference |
| --- | --- | --- | --- | --- |
| Mini-Mental State (MMSE) | General cognitive  impairment | | 28.73  (1.08) | (Folstein, 1975) |
| Trail Making Test (TMT) Form A & B | Executive functions: Task switching | Task switching costs for task completion times: B/A | 2.42 (0.67) | (Reitan, 1966) |
| Strooptest | Interference, inhibition | Interference costs for task completion times: incongruent/color | 1.8 (0.31) | (Spreen et al., 1998) |
| Verbal span (VeS) forwards | Verbal and spatial short term memory and working memory | Total number of correctly recalled items | 8.13 (1.98) | (Härting et al., 2000) |
| VeS backwards |  |  | 6.73 (2.1) |  |
| Visual span (ViS) forwards |  |  | 7.9 (1.9) |  |
| ViS backwards |  |  | 7.43 (1.87) |  |

**Supplementary Table 1.** Neuropsychological tests conducted with the healthy elderly participants.
